# Effects of acute intra-abdominal hypertension on multiple intestinal barrier functions in rats

**DOI:** 10.1038/srep22814

**Published:** 2016-03-16

**Authors:** Yuxin Leng, Min Yi, Jie Fan, Yu Bai, Qinggang Ge, Gaiqi Yao

**Affiliations:** 1 Department of Intensive Care Unit, Peking University Third Hospital, North Garden Road, No. 49, Haidian District, Beijing 100191, People’s Republic of China

## Abstract

Intra-abdominal hypertension (IAH) is a common and serious complication in critically ill patients for which there is no well-defined treatment strategy. Here, we explored the effect of IAH on multiple intestinal barriers and discussed whether the alteration in microflora provides clues to guide the rational therapeutic treatment of intestinal barriers during IAH. Using a rat model, we analysed the expression of tight junction proteins (TJs), mucins, chemotactic factors, and Toll-like receptor 4 (TLR4) by immunohistochemistry. We also analysed the microflora populations using 16S rRNA sequencing. We found that, in addition to enhanced permeability, acute IAH (20 mmHg for 90 min) resulted in significant disturbances to mucosal barriers. Dysbiosis of the intestinal microbiota was also induced, as represented by decreased Firmicutes (relative abundance), increased Proteobacteria and migration of Bacteroidetes from the colon to the jejunum. At the genus level, Lactobacillus species and Peptostreptococcaceae *incertae sedis* were decreased, whereas levels of lactococci remained unchanged. Our findings outline the characteristics of IAH-induced barrier changes, indicating that intestinal barriers might be treated to alleviate IAH, and the microflora may be an especially relevant target.

Intra-abdominal hypertension (IAH; sustained elevation in intra-abdominal pressure of 12 mmHg or above in adults and 10 mmHg or above in children), is a serious complication in critically ill patients, for which there are no well-defined treatment strategies^1^. Deterioration of IAH, resulting in sustained intra-abdominal pressure (IAP) of 20 mmHg or above, associated with new onset organ failure leads to ‘abdominal compartment syndrome’ (ACS)^1^,^2^. The mortality rate in patients with ACS may be as high as 50% due to multi-organ failure (MOF)^3^.

The intestinal mucosa serves as the first line of defence in protecting the host from enteric toxins and pathogenic microorganisms. In the 1990 s, Deitch *et al*. revealed the associations between mucosal defence mechanisms and the development of gut-derived sepsis^4^,^5^,^6^. They proposed that, “the activation of the intestinal mucosal immune system induced by bacterial translocation (BT) and barrier permeability changes may be the motor of MOF”^7^. Numerous studies have since confirmed the positive correlation between increased IAP and BT^8^,^9^,^10^,^11^,^12^,^13^. However, the characteristic IAH-induced intestinal barrier changes, especially changes in the barrier-associated microbiota, are not well understood.

Among the multiple intestinal barriers, a few studies have specifically investigated alterations to the mechanical barrier function of the intestine during IAH^11^,^14^,^15^, while changes in biologic, chemical and immune- barriers are lack of understanding. In addition to the epithelial cells of the intestine, the mucosal surface is coated with mucins, colonised by microflora, and is protected from certain noxious agents by a large number of lymphocytes. These components interact with each other to form an intact intestinal barrier and protect the gut against invasion by pathogens^16^,^17^,^18^,^19^,^20^. In the present study, we observed alterations in the diversity of intestinal microflora, as well as changes in the expression of TJ proteins (claudin 5, occludin 1), mucins (mucin 1, mucin 4, and mucin 2), chemotactic factors (MCP 1, CXCL 1, MIP 1β), and Toll-like receptor 4 (TLR4). Moreover, we tried to explain how the host-microbiota interaction becomes disturbed, and to provide clues for the rational therapeutic treatment of IAH.

## Results

### Effect of acute IAH on intestinal permeability in a rat model

**Figure 1:**
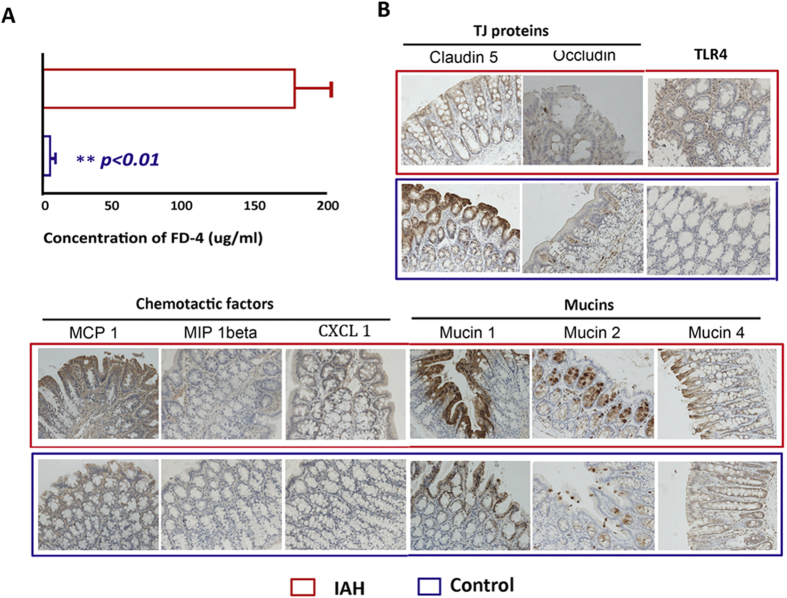
The effects of IAH on the intestinal permeability of FD-4 and expression of TJ proteins, mucins, chemotactic factors, and TLR4. (**A**) Intestinal permeability. Elevation of IAP to 20 mmHg resulted in a significant increase in concentration of FD-4 in portal plasma. (**B**) Expression of TJ proteins, mucins, chemotactic factors, and TLR4 (×200). Acute IAH led to a decrease in the expression of claudin 5, occludin and an increase in the expression of mucins, chemotactic factors, and TLR4. Statistical analysis on intestinal permeability was performed by independent-samples t-test. ^**^P < 0.01.

Intestinal permeability to macromolecules was evaluated by measuring FITC-dextran (FD-4, molecular weight 4000 Da) leakage from the gut cavity into the portal circulation. As shown in Fig. 1A, almost no FD-4 was detected in the portal venous system (5.3 ± 1.8 μg/mL) of control rats, while in rats with IAH, 20 mmHg-IAP resulted in a significant increase (*P* < 0.01) in the concentration of FD-4 in the plasma of portal blood (178.2 ± 26.0 μg/mL).

### 2.2 Effect of acute IAH on the expression of TJ proteins, mucins, chemotactic factors and TLR4

The expression of TJ proteins, mucins, chemotactic factors, and TLR4 was analysed by immunohistochemistry to investigate the influence of acute IAH on mechanical, chemical and immune barriers. Exposure to experimental pneumoperitoneum (20 mmHg, N_2_) for 90 min affected the staining intensity, as well as the percentage of cells staining for TJ proteins, mucins, chemotactic factors, and TLR4 (Fig. 1B). A significant difference was observed in the immunoreactive score (IRS) for most of the investigated factors between the IAH group and control group (Table 1). The IAP of 20 mmHg led to marked decreases in claudin 5 and occludin, which are the major TJs responsible for TJ permeability and paracellular transport^21^. In contrast, the IAP of 20 mmHg resulted in a significant increase in the levels of mucin 1, mucin 2, CXCL 1, MIP 1beta, MCP 1, and TLR4. The levels of these factors correlate closely with the disruption of chemical and immune barriers.

### IAH-induced changes in intestinal microflora

#### MiSeq sequencing results

**Figure 2:**
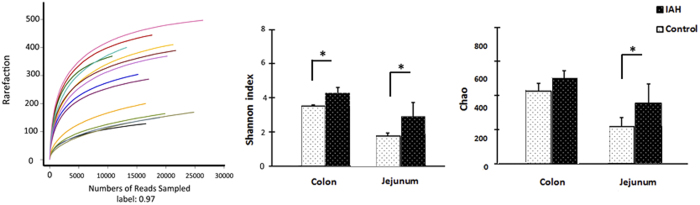
Rarefaction and estimators. Both the Shannon index and Chao index in the jejunum of IAH animals were markedly higher than in controls. In the colon, the two parameters of IAH rats were higher, although the difference in the Chao index was not statistically significant (*P* = 0.077). All the values are given as the mean ± SD. Statistical comparisons were performed by Mann-Whitney test. ^*^P < 0.05.

A total of 286,590 valid reads were obtained from 14 samples by MiSeq sequencing (n = 4 for content of colon and jejunum in IAH, n = 3 for content of colon and jejunum in control). Rarefaction curves and estimators are shown in Fig. 2. The rarefaction curves tended to approach the saturation plateau, indicating that the sequencing was deep enough to capture most of the OTUs within our samples. The Chao and Shannon indices of the IAH group were significantly higher than those of the control group. This demonstrates that exposure to 20 mmHg nitrogen pneumoperitoneum for 90 min resulted in an increase in both the microflora community richness and the microflora community diversity in rats.

#### Effect of acute IAH on principal component analysis (PCA) scores

**Figure 3:**
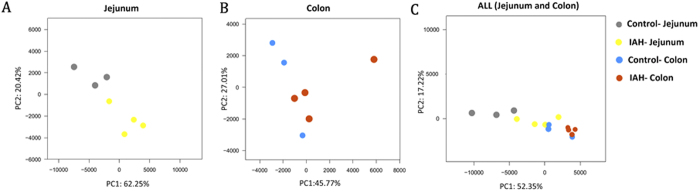
PCA plots PCA plots of intestinal microflora under the influence of IAH, based on unweighted ‘unifrac’ metrics. Each symbol represents a sample. (**A**) Microflora of the jejunum. The first two PCA scores were 62.25% and 20.42%, accounting for a total variation of 82.67% between the IAH and control group. (**B**) Microflora of the colon. The first two PCA scores were 45.77% and 27.01%, accounting for a total variation of 72.78% between IAH and control groups. (**C**) Microflora of the jejunum and colon. The combined data from the colon and the jejunum. The symbols identify four groups that can be separated.

The PCA score plots showed significant differences in the microfloral structure of the intestinal content between rats with IAH and controls (Fig. 3). In the jejunum, the first two PCA scores were 62.25% and 20.42% respectively, accounting for 82.67% of the variation between the two groups. Similar results were found in the colon. PC1 and PC2 were 45.77% and 27.01% respectively. When the data from all samples (including the IAH- Jejunum, IAH-Colon, Control- Jejunum, Control- Colon) were analysed together, primary differences of microfloral structure could still be detected among the four groups.

#### Changes in the microbial composition during IAH

**Figure 4:**
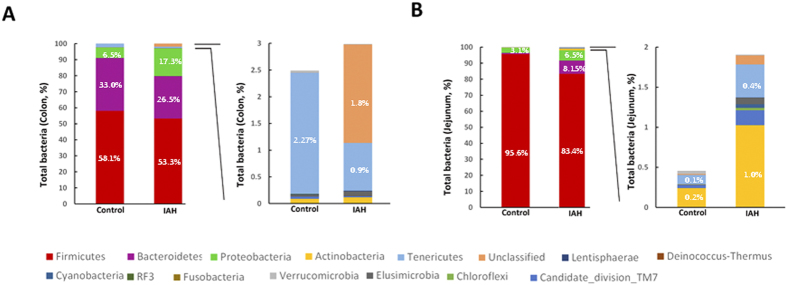
The influence of IAH on microbial composition at the phylum level. (**A**) Colonic microbial composition. (Left) Percentage of bacterial phyla in control and IAH rats. (Right) Bacterial phyla representing less than 5% of the total bacteria in controls and rats with IAH. (**B**) Jejunal microbial composition. (Left) Percentage of bacterial phyla in controls and IAH rats. (Right) Bacterial phyla representing less than 3% of the total bacteria in controls and rats with IAH. Sequences that could not be classified into any known group were designated as ‘Unclassified’. Statistical comparisons were performed by the Mann-Whitney test.

The patterns seen in microbial composition were quite dissimilar in the IAH experimental group and in controls. At the phylum level, the microflora of the jejunum and colon in the controls were dominated by species of the phyla Firmicutes, Bacteroidetes, and Proteobacteria. IAH did not alter the dominant phyla inhabiting either the colon or jejunum. However, an alteration in the relative abundance (RA) of these three phyla was observed in rats with acute IAH. IAH resulted in a decrease in the RA of Firmicutes species (IAH *vs*. Control, jejunal content: 83.4% *vs*. 95.6%; colonic content: 53.3% *vs*. 58.1%) and an increase in the RA of Proteobacteria (IAH *vs*. Control, jejunal content: 6.5% *vs*. 3.1%, *P* < 0.05; colonic content: 17.3% *vs*. 6.5%, *P* < 0.05) in both the jejunum and colon (Fig. 4). The influence of IAH on Bacteroidetes species was different in distinct segments of the intestinal tract. Exposure to 20 mmHg nitrogen pneumoperitoneum for 90 min resulted in a decrease in the RA of Bacteroidetes species in the colon (IAH *vs*. Control: 26.5% *vs*. 33.0%, *P* < 0.05) and an increase in the RA of Bacteroidetes species in the jejunum (IAH *vs*. Control: 8.15% *vs*. 0.01%, *P* < 0.05).

**Figure 5:**
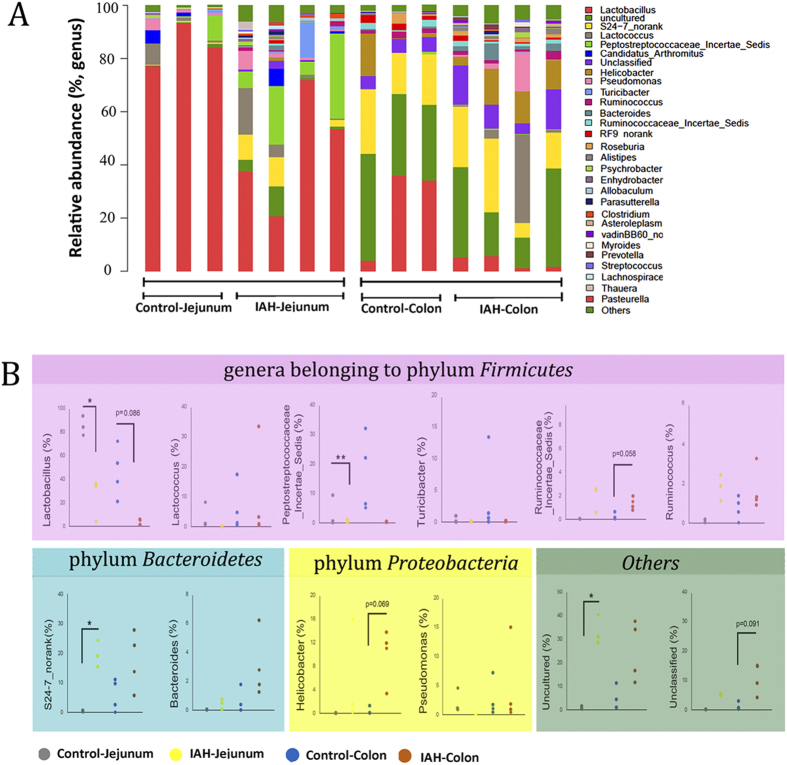
The influence of IAH on microbial composition at the genus level. (**A**) Distribution of bacterial taxa in jejunal and colonic content. (**B**) Comparison of RA of OTUs. There is a greater abundance in rats with IAH, compared to controls. Sequences that could not be classified into any known group were designated as ‘Unclassified’. Statistical comparisons were performed by the Mann-Whitney test. *P < 0.05, **P < 0.01.

The influence of IAH on the RA of the intestinal microflora at the genus level is shown in Fig. 5A. The impact of IAH on the twelve relatively abundant microflora genera was statistically analysed (Fig. 5B). Half of the species among these genera belonged to the phylum Firmicutes (*Lactobacillus, Lactococcus*, Peptostreptococcaceae *incertae sedis, Turicibacter*, Ruminococcaceae *incertae sedis* and *Ruminococcus*), two belonged to the phylum Bacteroidetes *(S24-7 no rank* and *Bacteroides)*, and two belonged to the phylum Proteobacteria *(Helicobacter* and *Pseudomonas)*. Amongst the Firmicutes species, IAH resulted in a downregulation of lactobacilli (IAH *vs*. Control: Colon: 3.5 ± 3.0% *vs*. 46.0 ± 18.9%; Jejunum: 24.7 ± 19.1% *vs*. 84.9 ± 8.1%) without any apparent impact on lactococci (Fig. 5B). In addition, the Peptostreptococcaceae *incertae sedis* load in the jejunum was reduced by IAH (IAH *vs*. Control: 0.4 ± 0.6% *vs*. 3.3 ± 5.2%). An increase in the abundance of Ruminococcaceae *incertae sedis* and *Ruminococcus* species was observed in rats with acute IAH, although the differences were not significant. The RA of *Helicobacter*, *Pseudomonas, Bacteroides*, and *S24-7_no rank* species increased.

## Discussion

The damage to the barrier functions of the gastrointestinal (GI) tract, triggered by IAH associated ischaemia and subsequent oxidative injury, is considered a likely pathophysiological basis for the IAH-associated multi-organ failure (MOF) cascade^15^,^22^. TJ proteins, mucins, TLRs, chemotactic factors, and the intestinal microflora are all representative components of the host-microbiota interaction^16^,^23^,^24^,^25^. Any disturbance to the intestinal homeostasis maintained by the synergistic activity of these factors with the GI cells can trigger GI dysfunction, including gut-derived sepsis. We proposed that in cases of IAH, pathogens within the deregulated GI microflora might cross the mucin-containing mucus layer, invade the epithelial cell lining through disrupted TJs, and then interact with TLRs to elicit or magnify innate immune responses, or migrate directly to blood vessels and mediate sepsis^26^. However, until this study was performed our reasonable speculations lacked evidence.

In this study, we found that, accompanying enhanced permeability, acute IAH (20 mmHg for 90 min) resulted in significant disturbances to multiple intestinal barriers, together with a marked decrease in the immunoreactivity of TJ proteins and a significant increase in the immunoreactivity of mucin 1, mucin 2, CXCL 1, MIP 1beta, MCP 1, and TLR4 (Fig. 1). Dysbiosis of the intestinal microbiota was also induced, as demonstrated by decreased levels of Firmicutes (relative abundance), increased Proteobacteria and migration of Bacteroidetes from the colon to the jejunum. At the genus level, although lactococci levels remained unchanged, the levels of lactobacilli and peptostreptococcaceae *incertae sedis* significantly decreased.

Among the markers of distinct intestinal mucosal barriers investigated in this study, only the changes of TJs under IAH had been previously investigated, to the best of our knowledge^11^,^14^,^15^. TJs are known to regulate the mechanical permeability of tissues by securing adjacent cells and forming mechanical barriers against extracellular fluids and materials^27^,^28^. Cheng *et al*. reported that a reduction in intestinal microcirculatory blood flow (MBF) was associated with disruption of the ultrastructure of TJs, resulting in marked dilation between adjacent epithelial cells in animals with IAH^11^,^14^. These data are consistent with our findings in this study for claudin 5 and occludin 1. Optimum mucus activation and normal mucosal immune functioning are important for proper regulation of intestinal inflammation^29^,^30^. Chang *et al*. reported that complete ischaemia in the splanchnic artery of rats for 30 min, resulted in breakdown of intestinal mucin accompanied by increased intestinal permeability to FITC-dextran, as well as the degradation of TLR4^29^. Bacterial penetration facilitated by loss of the mucus barrier, as well as disturbed mucosal immune functioning, can be rapidly counteracted by increased goblet cell secretory activity^30^. In contrast, in our nitrogen pneumoperitoneum-induced IAH model, the impact of IAH on mucins and TLR4 was quite different. We observed an upregulation of mucins and TLR4, accompanied by overexpression of chemotactic factors. We speculate that this may be due to an attempt at strong GI self-repair, coincident with dysregulated immune signalling.

Not only does the intestinal microbiota act as biological barrier, but it also participates in the formation of other three barriers. In addition, an imbalance in the microbial communities can provide a higher load of bacterial antigens^26^. Once the gut becomes leaky, the intestinal mucosa tends to become damaged, providing opportunities for the invasion of pathogens^31^. Microfloral transplants, or interventions with probiotics, have recently been reported to be effective in maintaining mucosal barrier integrity and suppressing the development of sepsis in critically ill patients^32^,^33^. In this process, dysbiosis is corrected and the TJ proteins and colonic mucins are upregulated, restoring healthy barrier function^33^,^34^. These striking findings provide new inspiration in IAH intervention with probiotics. As we demonstrated in this study, Lactobacillus species, well-established probiotics, become significantly decreased during an experimentally produced 90 min nitrogen pneumoperitoneum at 20 mmHg. Nevertheless, those genera potentially responsible for various intra-abdominal infections^35^, including Ruminococcaceae *incertae sedis, Ruminococcus, Helicobacter, Pseudomonas, Bacteroides and S24-7_no rank* species, were all shown to increase in abundance.

The specific mechanisms by which IAH disturbs the interaction between host immunity and the microbiota needs further investigation. With reference to other diseases, we speculate that the changes in microbial composition detected in the present study might be initiated by oxidative damage, and may be the consequence of subsequent disturbance to the local immune functioning^15^,^36^. A wide range of immune effector proteins (Toll-like receptors, Nod-like receptors, cytokines, IgA antibodies, anti-bacterial lectins (such as RegIIIγ)), and immune cells (natural killer T cells, dendritic cells, and mucin secreting T helper cells) may all be adversely influenced, as reported in the literature^37^,^38^,^39^. Lactobacilli, as well-established probiotics, can attenuate abnormalities in intestinal barriers by eliciting Treg cell differentiation, leading to expression of anti-inflammatory cytokines, by promoting discrimination of pathogenic bacteria by macrophages *via* interferon-mediated TLR gene regulation, and by interacting with specific receptors on dendritic cells. The effectors involved may include lipoproteins, glycolipids, bacterial short chain fatty acids, bacterial proteases, and certain cell surface proteins such as S-layer proteins^39^,^40^,^41^,^42^,^43^,^44^,^45^,^46^,^47^. These previously reported findings bolster our findings of intestinal barrier abnormalities upon IAH.

In conclusion, we characterised the dysfunctions of intestinal barriers during acute IAH. Upon production of acute IAH, we observed a striking alteration in the microbial composition of the GI tract. The disturbed host-microbiota interactions produced conditions that could, in principle, result in gut-derived sepsis. Our results in this animal model strengthen the clinical case for restoring intestinal barrier function, perhaps using probiotics (like lactobacilli), to treat critically ill patients that develop IAH.

## Materials and Methods

### Animals

To study the effects of acute IAH on intestinal barrier functions, 21 male SPF Sprague-Dawley rats (8-weeks-old, weight 200–250 g) were randomly assigned to the IAH group (20 mmHg, n = 12), or control group (n = 9). The rats in each group were further randomly assigned to three subgroups, for the detection of intestinal permeability to macromolecules, assessing microflora community diversity, and characterising the immune and chemical barrier functions (n = 4 per group for IAH, n = 3 per group for control). All experimental procedures, including the care and handling of animals, were performed following international guidelines. (Guide for the Care and Use of Laboratory Animals, Institute of Laboratory Animal Resources, Commission on Life Sciences, National Research Council, National Academy Press, Washington, DC, USA, 1996.)^48^ The rationale, design, and protocols for our study were approved by the Peking University Biomedical Ethics Committee-Experimental Animal Ethics Branch (Approval No. LA2013-12), prior to the initiation of experiments. The rats were housed solitarily in polypropylene cages and kept under standard controlled environmental conditions with 12 h light/dark cycles. The rats had free access to standard rat chow and water, which were autoclaved before use. The rats were deprived of food but not water for 12 h before inducing nitrogen pneumoperitoneum for 90 min (described below).

All surgeries were conducted under sodium pentobarbital anaesthesia (intraperitoneal injection, 40 mg/kg) and all efforts were made to minimise pain. After the procedure was complete, the animals were euthanized with an overdose of sodium pentobarbital (intraperitoneal injection, 160 mg/kg) to minimise pain^49^,^50^.

### Establishment of rats with acute IAH

The acute IAH animal model was established using the 90 min-nitrogen pneumoperitoneum procedure, which was previously described^15^. Briefly, after anaesthesia with an intraperitoneal injection of sodium pentobarbital (40 mg/kg), the rats were placed supine in a restraining apparatus, on a heated operating table to maintain their body temperature at 37 °C. Nitrogen pneumoperitoneum was performed by injecting nitrogen using a disposable venous infusion needle connected to a micro infusion pump. The micro infusion pump was linked to a blood pressure meter to dynamically monitor the IAP. When the target IAP was achieved, a low flow of nitrogen (1 mL/h) was used to maintain the desired IAP. In this study, the target IAP was 20 mmHg. The control animals were treated similarly, but no nitrogen was injected.

### Assessing intestinal permeability to FD-4 macromolecules

The intestinal permeability to FD-4 (molecular weight 4000 Da; Sigma-Aldrich, St. Louis, MO, USA, Product No. 68059) was assessed as previously described^15^. Briefly, after anaesthesia, a 10-cm segment of the distal ileum with preserved superior mesenteric vessels was dissected 3 cm proximal to the caecum. One millilitre of phosphate-buffered saline (0.1 mol/L, pH 7.2) containing 25 mg of FD-4 was injected into this ligated 10-cm intestinal lumen and the lumen was carefully replaced in the abdomen, covered, and protected with gauze soaked in warm saline. After 30 min, portal venous blood samples were collected to analyse the FD-4 concentrations spectrophotometrically at an excitation wavelength of 492 nm and an emission wavelength of 518 nm.

### Sample collection

After 90 min, the nitrogen pneumoperitoneum was decompressed gradually and laparotomy was performed by median abdominal incision. The samples of jejunal and colonic tissues (1 cm × 1 cm) were collected and gently flushed using cold saline. The tissue samples were then fixed in 10% neutral buffered formalin (pH 7.4) for immunohistochemical analyses. For detection of microfloral diversity, 10-cm long jejunal and colonic segments were isolated, and their contents were collected and stored in sterile freezing tubes. The samples were frozen by immersion in liquid nitrogen and stored at −80 °C until needed for experiments.

### Immunohistochemical staining and evaluation

Tissue preparation and immunohistochemical staining for TJ proteins, Toll like receptor 4, chemotactic factors, and mucins was performed as previously described^51^. Briefly, the tissue was incubated with primary antibodies (Table 2) overnight at 4 °C, washed, and analysed using goat anti-rabbit (PV-6001, Zhongshan Golden Bridge, Beijing, China) or anti-mouse (PV-6002, Zhongshan Golden Bridge) detection kits. Micrographs were obtained (Nikon E600, Nikon, Tokyo, Japan) to determine the immunoreactive score (IRS). IRS was defined as the product of staining intensity (SI) and the percentage of positive cells (PP)^52^. The details of the scoring system are described in Table 3. Scoring was performed by three blinded observers. Briefly, five different fields (at ×200 magnification) in each slide were selected and analysed and the mean value of the measurements by the three observers was recorded as the final result. Details of the primary antibodies are given in Table 2.

### Analysis of Intestinal Microflora Community Diversity

#### DNA Extraction, PCR amplification, and Illumina MiSeq sequencing

##### DNA Extraction and PCR amplification

Microbial DNA was extracted using the E.Z.N.A. stool DNA Kit (Omega Bio-tek, Norcross, GA, USA) according to the manufacturer’s protocols. The V4-V5 region of the bacterial 16S ribosomal RNA gene were amplified by PCR (95 °C for 2 min, followed by 25 cycles at 95 °C for 30 s, 55 °C for 30 s, 72 °C for 30 s. A final extension at 72 °C for 5 min, was included). The primers used were 338F 5′-barcode- GTGCCAGCMGCCGCGG)-3′ and 806R 5′-CCGTCAATTCMTTTRAGTTT-3′: barcode is an eight-base sequence unique to each sample. All PCR reactions were performed in triplicate with 20 μL final reaction mixture containing 4 μL of 5× Fast Pfu Buffer, 2 μL of 2.5 mM dNTPs, 0.8 μL of each primer (5 μΜ), 0.4 μL of FastPfu Polymerase, and 10 ng of template DNA.

##### Illumina MiSeq sequencing

Amplicons were extracted from 2% agarose gels and purified using the AxyPrep DNA Gel Extraction Kit (Axygen Biosciences, Union City, CA, USA) according to the manufacturer’s instructions, and quantified using QuantiFluor-ST (Promega, USA). The purified amplicons were pooled in equimolar quantities and paired-end sequenced (2 × 250) on an Illumina MiSeq platform according to standard protocols.

#### Processing of sequencing data and data analysis

Raw ‘fastq’ files were demultiplexed and quality-filtered using *QIIME* (version 1.17) with the following criteria: (i) Reads with 250 bp, truncated at any site, received an average quality score <20 over a 10-bp sliding window. Reads shorter than 50 bp were discarded. (ii) For exact barcode matching, reads with two nucleotide mismatches in primer matching. Reads containing ambiguous characters, were discarded. (iii) Sequences that had overlaps longer than 10 bp were assembled according to their overlap sequence. The reads that could not be assembled were discarded. Sets of sequences with ≥97% identity were defined as an Operational Taxonomic Unit (OTU) using *UPARSE* (version 7.1 http://drive5.com/uparse/). Any chimeric sequences were identified and removed using *UCHIME*. The phylogenetic affiliation of each 16S rRNA gene sequence was analysed by *RDP Classifier* (http://rdp.cme.msu.edu/) against the SILVA 16S rRNA database (SSU115, Max Planck Institute, Germany) using a confidence threshold of 70%^53^,^54^.

Rarefaction curves, alpha diversity (Shannon, Chao), and beta diversity calculations were performed using *QIIME*. Unweighted UniFrac distance-metrics analysis was performed using OTUs for each sample^55^,^56^. Principal component analysis (PCA) was then performed based on matrix-of-distance. To analyse the influence of IAH on microfloral diversity, comparisons between IAH-J and Control-J, IAH-C and Control-C were conducted using the Mann-Whitney test at the level of phyla, and genera at 97% OTU levels^57^.

### Statistical Analysis

The Mann-Whitney test and independent t-test were performed using *SPSS* 16.0 software (SPSS, Chicago, IL, USA). *P*-values < 0.05 were considered statistically significant.

## Additional Information

**How to cite this article**: Leng, Y. *et al*. Effects of acute intra-abdominal hypertension on multiple intestinal barrier functions in rats. *Sci. Rep*. **6**, 22814; doi: 10.1038/srep22814 (2016).

## Figures and Tables

**Table 1:** IRS scores of TJ proteins, mucins, chemotactic factors, and TLR4.

Intestinal segment		Claudin 5	Occludin 1	Mucin 1	Mucin 2	Mucin 4	MCP1	CXCL 1	MIP 1beta	TLR4
Jejunum	IAH	2.73 ± 1.32**	0.90 ± 0.45*	5.55 ± 3.07**	5.95 ± 3.96**	2.76 ± 2.03	6.66 ± 4.61**	2.99 ± 2.12**	2.33 ± 1.72**	3.99 ± 3.23**
	Control	4.98 ± 2.77	1.32 ± 1.23	2.84 ± 1.93	1.97 ± 1.83	2.07 ± 1.30	3.58 ± 2.12	0.88 ± 0.69	1.35 ± 0.82	0.84 ± 0.67
Colon	IAH	2.46 ± 1.52**	0.78 ± 0.64**	5.86 ± 4.01**	5.58 ± 4.06**	2.68 ± 2.50	6.45 ± 3.61**	2.80 ± 2.52**	1.93 ± 1.76**	3.89 ± 3.33**
	Control	5.20 ± 3.51	1.53 ± 1.28	2.04 ± 1.77	1.77 ± 1.75	1.97 ± 1.70	3.01 ± 2.42	0.94 ± 0.89	0.87 ± 0.75	0.69 ± 0.67

All the values are given as the mean ± SD. Statistical comparisons between the IAH group and the control group were performed by an independent-samples t-test. *P < 0.05, **P < 0.01.

**Table 2:** Details of the antibodies used.

Antibody	TLR4	Claudin 5	Occludin 1	Mucin 1	Mucin 2	Mucin 4	MCP 1	CXCL 1	MIP 1beta
Company	Abcam	Millipore	Invitrogen	Abcam	Abcam	Invitrogen	Abcam	Abcam	Abcam
Product Number	ab30667	ABT45	1204426A	ab45167	ab134119	35–4900	Ab7202	ab86436	Ab25129
Source	Mouse	rabbit	mouse	rabbit	rabbit	mouse	rabbit	rabbit	rabbit
Dilution	1:200	1:200	1:400	1:200	1:250	1:200	1:100	1:200	1:200

**Table 3:** Details of the immunoreactive scoring system.

		SI [0–3]			
IRS [SI × PP]		*Negative*	*Weak (1)*	*Moderate (2)*	*Strong (3)*
PP [0–4]	*Negative (0)*	0	0	0	0
	≤*10% (1)*	0	1	2	3
	≥*11%*, ≤*50% (2)*	0	2	4	6
	≥*51%*,≤*80% (3)*	0	3	6	9
	≥*81% (4)*	0	4	8	12

IRS, immunoreactive score; SI, staining intensity; PP, the percentage of positive cells.
